# Government drivers of breast cancer prevention: A spatiotemporal analysis based on the association between breast cancer and macro factors

**DOI:** 10.3389/fpubh.2022.954247

**Published:** 2022-10-04

**Authors:** Xiaodan Bai, Xiyu Zhang, Hongping Shi, Guihong Geng, Bing Wu, Yongqiang Lai, Wenjing Xiang, Yanjie Wang, Yu Cao, Baoguo Shi, Ye Li

**Affiliations:** ^1^Department of Economics, School of Economics, Minzu University of China, Beijing, China; ^2^Research Center of Health Policy and Management, School of Health Management, Harbin Medical University, Harbin, China; ^3^Department of Oncology, Heze Municipal Hospital, Heze, China

**Keywords:** breast cancer scale, light at night, macro factors, geographically and temporally weighted regression model, temporal and spatial heterogeneity

## Abstract

**Background:**

Currently, breast cancer (BC) is ranked among the top malignant tumors in the world, and has attracted widespread attention. Compared with the traditional analysis on biological determinants of BC, this study focused on macro factors, including light at night (LAN), PM2.5, per capita consumption expenditure, economic density, population density, and number of medical beds, to provide targets for the government to implement BC interventions.

**Methods:**

A total of 182 prefecture-level cities in China from 2013 to 2016 were selected as the sample of the study. The geographically and temporally weighted regression (GTWR) model was adopted to describe the spatiotemporal correlation between the scale of BC and macro factors.

**Results:**

The results showed that the GTWR model can better reveal the spatiotemporal variation. In the temporal dimension, the fluctuations of the regression coefficients of each variable were significant. In the spatial dimension, the positive impacts of LAN, per capita consumption expenditure, population density and number of medical beds gradually increased from west to east, and the positive coefficient of PM2.5 gradually increased from north to south. The negative impact of economic density gradually increased from west to east.

**Conclusion:**

The fact that the degree of effect of each variable fluctuates over time reminds the government to pay continuous attention to BC prevention. The spatial heterogeneity features also urge the government to focus on different macro indicators in eastern and western China or southern and northern China. In other words, our research helps drive the government to center on key regions and take targeted measures to curb the rapid growth of BC.

## Introduction

Cancer is the killer of human life (1). With the rapid development of medical technology, human beings are still unable to eliminate the pain of cancer and loss of life (2, 3). In all patterns of cancer, breast cancer (BC) incidence and mortality both lie on top of malignant tumors (4, 5). According to the International Agency for Cancer Research (IARC) of the World Health Organization (WHO), BC morbidity (11.7%) ranked first and mortality (6.9%) ranked fifth among all cancers in 2020 (6); BC has been a heavy burden on the global population (7). Additionally, the metastasis of BC cells will lead to the pathology of other organs (8, 9). Patients suffer from both physical pain and mental depression (10); thus the demand for health and longevity cannot be satisfied (11, 12). The exact carcinogenic factors of BC are not yet clear (13), so reducing the disease risk also faces difficulties. Today, the whole world is focusing on “converging attacks” from the two aspects of prevention and treatment of BC (14, 15) to break through the “BC dilemma.” In this study, an in-depth exploration and multidimensional analysis of the potential macro influences on BC provides a theoretical evidence-based basis for BC intervention.

Based on the theory of social determinants of health, the occurrence of disease is the result of multidimensional factors. Currently, the analyses of carcinogenic factors of BC focus on the following three aspects: congenital inheritance (5, 15–18), lifestyle and psychological pressure (15, 19–22), and physical environment (natural environment and social environment, etc.) (15, 23–26). In addition, the mechanism by which light at night (LAN) blocks melatonin formation and triggers BC has attracted much attention (27–33). Xiao et al. used the Cox proportional hazards models to estimate the hazard ratio of BC and found that different tumor stages and ethnic differences would cause different results in the effect of LAN on BC. In black women, the relationship between LAN and increased BC risk was observed for localized BC only, whereas in white women, the relationship was observed for regional/distant stages (32). Al-Naggar et al. applied a linear regression method to demonstrate a significant association between artificial light at night and diseases such as BC in protected areas (27). Lamphar et al. studied 25,025 breast cancer cases and found that cumulative light pollution was positively associated with BC and persisted after age standardization (31). These studies fully demonstrate the important role of remote sensing light in BC.

With the continuous innovation of research methods, the research analyzing the spatial characteristics of cancers has become more and more abundant. This is the key to taking targeted preventive measures in different regions based on the differential distribution of diseases in space, and is also the basis for efficient promotion of human health. Amin et al. used SaTScan software to identify significant BC spatial clusters in the United States and propose high emphasis on areas of spatial clustering of BC (34). Using the geographically weighted regression (GWR) method, in the study by Pes et al., a hotspot of gastric cancer mortality was detected in the central mountainous area of Sardinia among males, positively associated with goiter, and the practice of sheep-rearing, whereas there was a negative association with the diet score (35). Imounga et al. examined spatial trends in cervical cancer in French Guiana, reminding policymakers to focus on remote areas (36).

There is an increasing number of studies on the determinants of BC, but there are still some limitations. First, the research scale is mostly focused on the national scope, while the spatial distribution characteristics of BC are not described more precisely and are not conducive to targeted intervention. Second, although these studies have considered light impacts on BC, they pay more attention to the behavioral habits and physiological characteristics at the individual level. The study of the impacts of LAN on BC under the economic level, medical condition, and air quality lacks attention. However, macro factors play significant roles. For example, people in developed countries tend to have a high intensity of LAN, leading to a higher incidence of BC. The BC incidence in developed countries (71.7/100 thousand) is higher than that in less developed countries (29.3/100 thousand) (4). Additionally, a comprehensive early examination schedule, reasonable medical service supply and appropriate late treatment plan can effectively reduce the morbidity and mortality of BC (37). Furthermore, air pollution from a high level of fine particulate matter will increase breast density and raise the risk of BC. The emission density of PM2.5 in downtown Atlanta is 4.6 times higher than that in rural Georgia, and the average incidence of BC is 16.62% higher than that in rural Georgia (38). Third, most articles use statistical methods to process panel data and lack attention to spatial heterogeneity and time span. This may reduce the estimation efficiency, biased results, and lack of continuous changes in the data, which is too different from the real situation of BC.

In such a context, our study has some outstanding innovations. Considering the importance of the scale effect on spatial research, avoiding the roughness of large scale research at the global-level and unobservable small scale studies at the county-level, this study adopted an intermediate scale in prefecture-level cities and focused on the impacts of macroeconomic indicators on BC from a sustainable development perspective. In addition, to emphasize a spatiotemporal perspective, this study applied the geographically and temporally weighted regression (GTWR) model (39). By constructing a spatiotemporal dependent stereo model, we can obtain more accurate results (40) and consequently display different factors attributed to BC from various regions, for further targeted intervention measures. This will provide a theoretical reference for the prevention of BC in countries or regions with high incidence and has great significance for promoting human health.

## Materials and methods

### Variable selection and data source

This study considered China's prefecture-level cities or municipalities directly under the central government as the research objects. After sorting out the data fully, 182 research units were retained. The specific sampling method can be described in three steps. First, among all prefecture-level cities in China, 216 cities with established cancer surveillance centers were screened. Second, to ensure the spatial continuity of the panel data, we took 2013 as the base year and deleted the surveillance centers added later. Therefore, 182 prefecture-level cities were retained. Third, we collected data for each explanatory variable, matched them with the above 182 prefecture-level cities, and thus finalized the 182 prefecture-level cities.

Additionally, we also verified the sample size to ensure rigor. The minimum sample size was calculated by applying G^*^power 3.1.9.7 developed by Heinrich Heine University Düsseldorf. We chose the two-sided *t*-test and the difference between two independent means (two groups) as the test type and measured variable. The significance level is 5%, and the test power is 95%. Table 1 shows that the minimum sample size is 105. It is much smaller than the 182 in this study. So, the sample size in this paper meets the requirements for research reliability.

**Table 1:** The test of sample size.

**Output parameters**	**Values**
Non-centrality parameter δ	3.6228448
Critical *t*	1.9714347
Df	208
Sample size group 1	105
Sample size group 2	105
Actual power	0.9501287

In the process of building the model, we selected the number of BC cases (the BC scale) as the explained variable. These data were from the *China Cancer Registry Annual Report (2013–2016)*, published in 2017–2020. In addition, considering the significant geographic differences in disease morbidity and mortality (41–44), it is only through appropriate spatial methods that the spatial heterogeneity of disease can be displayed thoroughly, and targeted policies can be made accordingly. Ignoring spatial heterogeneity can lead to many problems, such as loss of estimation efficiency, biased estimation, and saliency of errors. Therefore, we analyzed the factors related to the spatial distribution pattern of BC as follows:

1. Light at night. The light data is obtained through spatial technology, so it is easy to match the regional geographic location (31). Light changes alter our circadian rhythm, especially the normal cycle of melatonin. This leads to early menarche and elevated circulating estrogen and prolactin, sex hormones that increase the risk of BC (28, 30, 32, 45). We include the LAN data in the explanatory variables. It uses Visible Infrared Imaging Radiometer Suite Day/Night Band (VIIRS/DNB) image data, which is one of the ways to collect light images at night and it has higher spatial resolution and a wider radiation detection range. The data was obtained from the Chinese Research Data Services Platform (CNRDS).
2. Environmental pollution. Aromatic hydrocarbon receptors in polluted environments mediate the effects of many endocrine disruptors and have implications for BC in young or premenopausal women (46–48). There is little literature on the effects of environmental pollution on BC from a spatial perspective. Under the constraints of data availability, we finally chose “PM2.5” as a proxy variable for environmental pollution. PM2.5 can potentially affect breast density by interfering with the growth of breast cells and increasing the relative amount of fibrous tissue (48, 49), thereby greatly enhancing the risk of BC. The data was obtained from the atmospheric composition analysis group of Dalhousie University.
3. Economic development and wealth level. From a spatial perspective, economic development and wealth level vary by geographic location (50). Considering the availability of data and the quality of variables, “economic density” and “per capita consumption expenditure” are chosen as proxies for economic development and wealth levels. In general, the higher the level of economic development or the higher the economic and social status of people, the greater attention they pay to BC prevention and screening (51, 52). Additionally, BC prevention and treatment are at a high level in terms of supply and demand. This came from the *China City Statistical Yearbook (2014–2017)* or the *Economic and Social Development Statistical Bulletin (2013–2016)*.
4. Population. To integrate the characteristics of population and area, we chose “population density” as the proxy variable. The occurrence of BC ultimately manifests in the individual. When the population base is large, the possibility of BC increases (53). The data were obtained from the *China City Statistical Yearbook (2014–2017)* or the *Economic and Social Development Statistical Bulletin (2013–2016)*.
5. Medical resources and medical service level. Based on the availability of data, “number of medical beds” was used as a proxy variable. If a region has abundant medical resources and a high level of medical services, the screened BC cases are very close to the actual number of patients, and a relatively high BC scale would be detected (54). The data was obtained from the *China City Statistical Yearbook (2014–2017)*.
6. Education level. We chose “average years of education” and “number of students in higher education” for each region as proxy variables. Higher education groups are more likely to accept the relevant knowledge and treatment process of disease prevention (55). Therefore, the prevention and treatment of BC are more effective among them.
7. Political background. Each region has experienced unique changes thus far, and these political changes will also have a certain impact on BC (56).

We attempted to find relevant data on “education level,” but unfortunately, they are too scarce due to covering 182 prefecture-level cities from 2013 to 2016. So, in the end, we have to exclude this factor. In addition, political factors have little impact on BC in China from 2013 to 2016, and are not easy to quantify. The “political background” was also excluded.

Stata, GeoDa, and ArcGis10.2 are adopted to process the data. Table 2 shows a specific description of these variables.

**Table 2:** Description of various variables.

**Variable**	**Interpretation**	**Unit**
The BC scale	Total number of male and female BC cases in each region	Person
LAN	The sensors on the satellite can detect the light information of the earth at night, representing the data of human activities	DN total value/number of grids
PM2.5	The higher the concentration of particles with aerodynamic equivalent diameter < 2.5 microns in the ambient air, the more serious the air pollution is	μg/m^3^
Permanent population	The population who often lives here or has lived here for more than 6 months throughout the year	10,000 people
Area size	Total area of a region	km^2^
GDP	The final value of production activities of all resident units in an area within 1 year	10,000 yuan
Per capita consumption	Total expenditure of residents to meet the daily consumption of families	Yuan/person
Economic density	GDP per unit area	100 million yuan/km^2^
Population density	Population per unit area	10,000 people/km^2^
Number of medical beds	The number of medical beds in each region, representing the medical resources of a region	1,000 sheets

### Research methods

#### Ordinary least squares (OLS)

The OLS model needs to select a set of linearly independent functions in advance, and obtain the closest result to the real situation by setting the undetermined coefficients and solving them. The condition of OLS is to use the least square method to obtain the unknown data and minimize the square sum of the error, that is, to minimize the square sum of the distance from all observations on the scatter diagram to the regression line. Its calculation formula is as follows:


$$y_{i}=\beta_{0}+\sum_{k}\beta_{k}x_{ik}+\varepsilon_{i}$$

In the formula, *i* is the prefecture level city number, *y*_*i*_ represents the BC scale in city i, *x*_*ik*_ represents the k-th explanatory variable of the i-th city, and β_0_ indicates the expected value of BC cases in different regions when all explanatory variables do not work. β_*k*_ is the k-th regression parameter of the control variable which indicates that the BC scale fluctuates with the change in explanatory variables. ε_*i*_ is a random error term.

#### Geographically weighted regression (GWR)

This method extends the traditional OLS from a global to a local framework by incorporating the spatial location into the parameters and using a locally weighted least squares method for point-by-point parameter estimation. The estimated parameters will change depending on geospatial location, thus visualizing the spatial heterogeneity of the study object. Its calculation formula is as follows (57):


$$y_{i}=\beta_{0}(u_{i},v_{i})+\sum_{k}\beta_{k}(u_{i},v_{i})x_{ik}+\varepsilon_{i}$$

(*u*_*i*_, *v*_*i*_) represents the centroid coordinates of city i. Unlike the spatial “fixed” coefficient estimates in the global model, this model allows the parameter estimates β_*k*_(*u*_*i*_, *v*_*i*_) to vary with space, so it can capture local effects. It is critical to select an appropriate weight matrix for estimating the parameters of GWR. The spatial weights can be estimated by a spatial kernel function, also called a distance-decay function. According to whether the bandwidth is varied, the 2 basic types of spatial kernels are fixed and adaptive kernels, which use fixed bandwidth and a fixed number of nearest neighbors within an adaptive bandwidth, respectively. Further, this method was chosen because quadratic kernel function had the best (smallest) AICc for fitting the GWR model to the data. So, this study selected the adaptive bandwidth quadratic kernel function commonly used in academia as the distance weight function. Its formula is:


$$w_{ij}=\{\begin{array}{cc} {\left[1-{\left(\frac{d_{ij}}{b_{i}}\right)}^{2}\right]}^{2} & if\;d_{ij}<b_{i}\\ 0 & otherwise \end{array}$$

Where *w*_*ij*_ is the weight of the impact of city i on city j, *d*_*ij*_is the distance between city i and city j, and *b*_*i*_ is the bandwidth specific to location i.

#### Geographically and temporally weighted regression (GTWR) and temporally weighted regression (TWR)

GTWR is an extended model of GWR. It not only considers the spatial non-stationarity of geographic data but also adds the time effect in the model to improve the goodness of fit of the model (58). Its formula is:


$$y_{i}=\beta_{0}(u_{i},v_{i}\;,t_{i})+\sum_{k}\beta_{k}(u_{i},v_{i}\;,t_{i})x_{ik}+\varepsilon_{i}$$

Where i(*u*_*i*_, *v*_*i*_, *t*_*i*_) represents the spatiotemporal coordinates of city i, *u*_*i*_, *v*_*i*_ represents the projected spatial coordinates, and *t*_*i*_ is the projected temporal coordinates. Unlike the global regression model with “fixed” coefficients, GTWR allows parameter estimation β_*k*_(*u*_*i*_, *v*_*i*_, *t*_*i*_) to vary across space and time. Therefore, this method can capture spatiotemporal changes at the same time. The estimated value of its parameters β_*k*_(*u*_*i*_, *v*_*i*_, *t*_*i*_) can be expressed as:


$${\hat{\beta }}_{k}(u_{i},v_{i}\;,t_{i})={\left[X^{T}W\left(u_{i},v_{i}\;,t_{i}\right)X\right]}^{-1}X^{T}W(u_{i},v_{i}t_{i})Y$$

where *W*(*u*_*i*_, *v*_*i*_, *t*_*i*_) is the space-time weight matrix, and its diagonal elements are the weight values of city i and its adjacent city j, GTWR defines *w*_*ij*_ as a weight matrix constructed by the adaptive Gaussian distance attenuation function. This makes the weight of data points closer to observation point i higher than that of data points farther from observation point i in a spatiotemporal coordinate system. In addition, in the GTWR model, the ${d_{ij}}^{ST}$ used in the weight matrix—the spatiotemporal distance between city i and city j—is defined as a linear combination of spatial and temporal distances:


$$d_{ij}{}^{ST}=\sqrt{\lambda \left[{\left(u_{i}-u_{j}\right)}^{2}+{\left(v_{i}-v_{j}\right)}^{2}\right]+\mu {\left(t_{i}-t_{j}\right)}^{2}}$$

Where λ and μ are the scale parameters of equilibrium space and time, respectively. In particular, when λ = 0, the spatiotemporal distance degenerates into the distance in the TWR model; when μ = 0, the spatiotemporal distance degenerates into the distance in the GWR model (59).

## Results

### Spatial variation characteristics of the BC scale from 2013 to 2016

**Figure 1:**
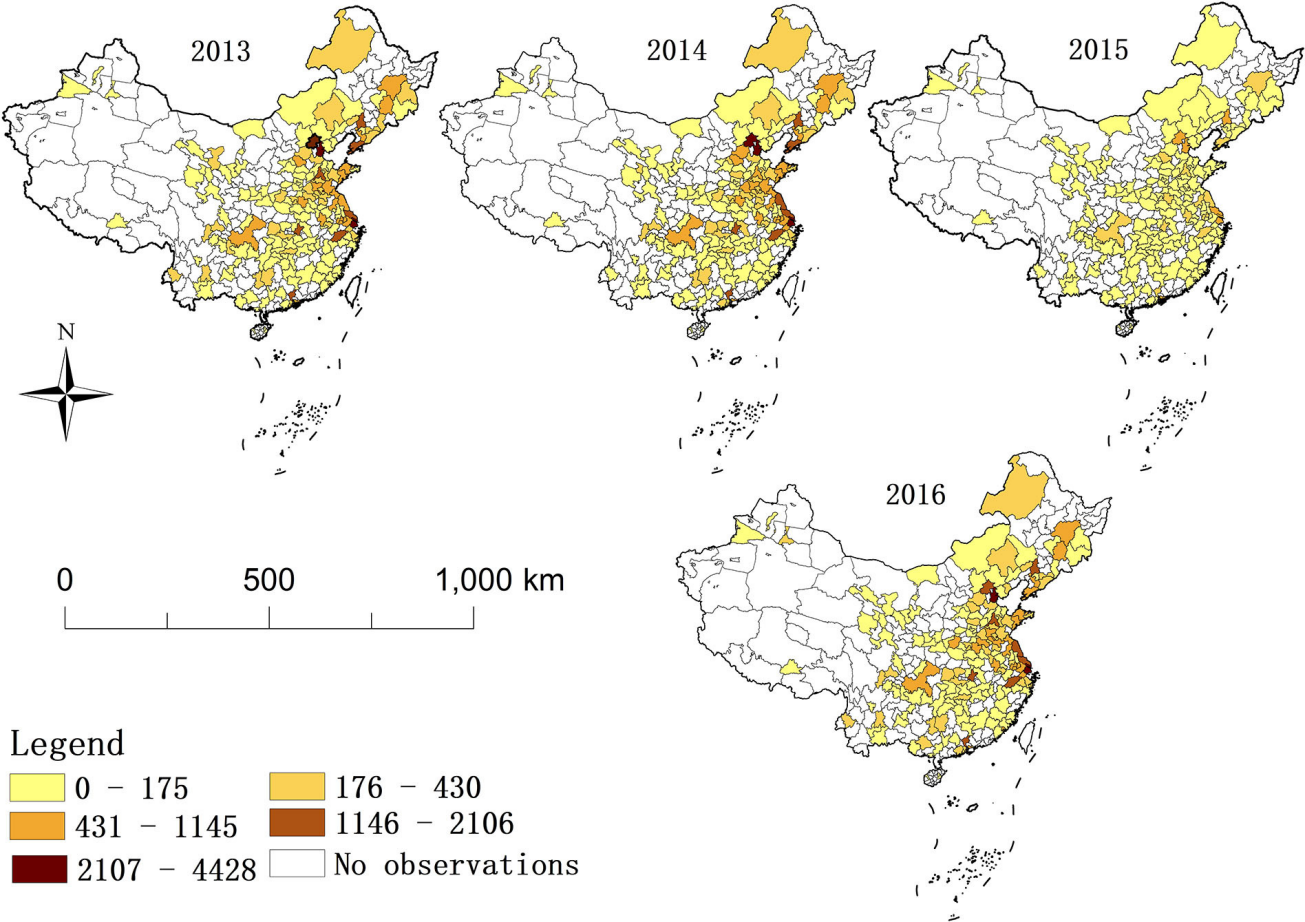
The scale of breast cancer in 182 Chinese prefectural-level units during 2013–2016.

The scale of BC in 182 prefectural-level units in China from 2013 to 2016 is shown in Figure 1. From a spatial perspective, the Liaodong Peninsula, Shandong Peninsula, and Beijing-Tianjin-Hebei area are located in the Bohai Rim and some cities in the Yangtze River Delta are areas with a high scale of BC. They also have a higher degree of BC clustering. Overall, there are eight prefecture-level cities with an average annual scale of BC exceeding 1,000. They are Beijing (2,597), followed by Tianjin (2,544), Shanghai (2,243), Hangzhou (1,550), Nantong (1,461), Shenyang (1,385), Guangzhou (1,223), and Wuhan (1,113). From the time perspective, compared with the former 2 years, the scale of BC in 2015 and 2016 is much lower, and 92.86% of prefecture-level cities have a lower scale of BC than the previous 2 years. In addition, the scale of BC in 2015 was lowest, so to reflect the continuous changes in the scale of BC more effectively, it was necessary to study both the spatial effects and time effects of the scale of BC.

### Results from OLS model

OLS regression is first used to explore the relationship of the scale of BC and LAN, PM2.5, per capita consumption expenditure, economic density, population density, and number of medical beds. This model can be used as a benchmark for comparison with local regression model results. Table 3 shows the estimated results of OLS. The *R*^2^ was 0.581, which indicated that OLS can explain at least 58.1% of the total variation in the scale of BC and has statistical significance. Additionally, the variance inflation factor (VIF) values were lower than 3, verifying that the choice of explanatory variables can avoid the problem of collinearity. According to the results in Table 3, the scale of BC had a strongly positive correlation with LAN, PM2.5, per capita consumption expenditure and number of medical beds (*p*-value < 0.1) and a negative correlation with population density (*p*-value < 0.05).

**Table 3:** Parameter estimate summaries of OLS on the entire data set.

**Variable**	**Co-ef**.	**St. err**.	***t*-statistic**	***P*-value**	**VIF**
Intercept	−84.312	30.924	−2.73	0.007***	–
LAN	12.862	6.671	1.93	0.054*	2.75
PM2.5	2.185	0.544	4.02	0.000***	2.45
Per capita consumption expenditure	13.586	6.262	2.17	0.030**	1.78
Economic density	10.771	9.177	1.17	0.241	1.53
Population density	−219.122	89.962	−2.44	0.015**	1.28
Number of medical beds	17.206	0.753	22.86	0.000***	1.1
*R* ^2^	0.581				
AICc	10,316.301				

*** *p* < 0.01,

** *p* < 0.05,

* *p* < 0.1.

### Results from GTWR model

Furthermore, the GTWR model was also adopted to verify the relationship between the scale of BC and the above explanatory variables. Compared with OLS, the fitting results of GTWR are significantly improved in terms of the *R*^2^ and AICc values. Table 4 shows five statistics, including each estimated parameter's minimum (Min), lower quartile (LQ), mean, upper quartile (UQ) and maximum (Max). For variable LAN, the Min and Max values of the coefficients are −6.161 and 44.114 respectively, indicating that the correlation between the scale of BC and LAN has obvious spatial-temporal variation. With LQ = 4.994 > 0, negative relationships exist for some spatial units or time frames, and positive relationships are dominant overall. Similarly, the coefficients of other variables also show apparent space-time variation; the differences between the maximum and minimum PM2.5, per capita consumption expenditure, economic density, population density, and number of medical beds are 4.489, 81.325, 236.969, 1,491.394, and 22.225, respectively.

**Table 4:** Parameter estimate summaries of GTWR on the entire data set.

**Variable**	**Min**	**LQ**	**Mean**	**UQ**	**Max**
Intercept	−167.39	−119.262	−84.366	−39.809	32.025
LAN	−6.161	4.994	21.169	34.296	44.114
PM2.5	−0.683	1.034	1.716	2.098	3.86
Per capita consumption expenditure	−12.122	5.918	23.018	45.439	69.203
Economic density	−209.127	−65.206	−35.143	12.93	27.824
Population density	−277.394	−231.967	94.174	239.197	1,214
Number of medical beds	4.136	14.823	17.509	24.077	26.361
*R* ^2^	0.747				
AICc	10,008.2				

### Performance comparison of different models

To illustrate the applicability of GTWR to this study, GWR and TWR are also tested on the same dataset, and Table 5 presents the fitting results of these models. The TWR model performs better than the GWR model, indicating that the fluctuation during 2013–2016 was greater than its spatial discrepancy. Namely, the time non-stationarity was greater than the spatial non-stationarity. GTWR exhibited the best performance, including the highest *R*^2^, lowest RSS and lowest AICc. It is worth mentioning that the comparison has two contributions to the whole thesis. It can prove that the GTWR model is more suitable for BC scale local effects. Also, our research content needs to eliminate one-by-one traditional methods (OLS, GWR, and TWR) and methodological upgrades. The research topic of this study was the temporal and spatial differences of the BC scale in 182 prefecture-level cities in China from 2013 to 2016 and the macro factors driving its changes, capturing both temporal and spatial local effects. Undoubtedly, this is something that a global model such as OLS cannot achieve. Therefore, this article selects the GTWR model to further describe the correlation between various influencing factors and the scale of BC.

**Table 5:** Performance comparison of four models on the entire data set.

	**OLS**	**GWR**	**TWR**	**GTWR**
Neighbor		235	237	244
RSS	59,645,963	45,486,100	37,244,200	36,034,100
*R*-square	0.5811	0.6811	0.7388	0.7473
AICc	10,316.301	10,180.6	10,021	10,008.2

### Temporal variation of estimated coefficients

**Figure 2:**
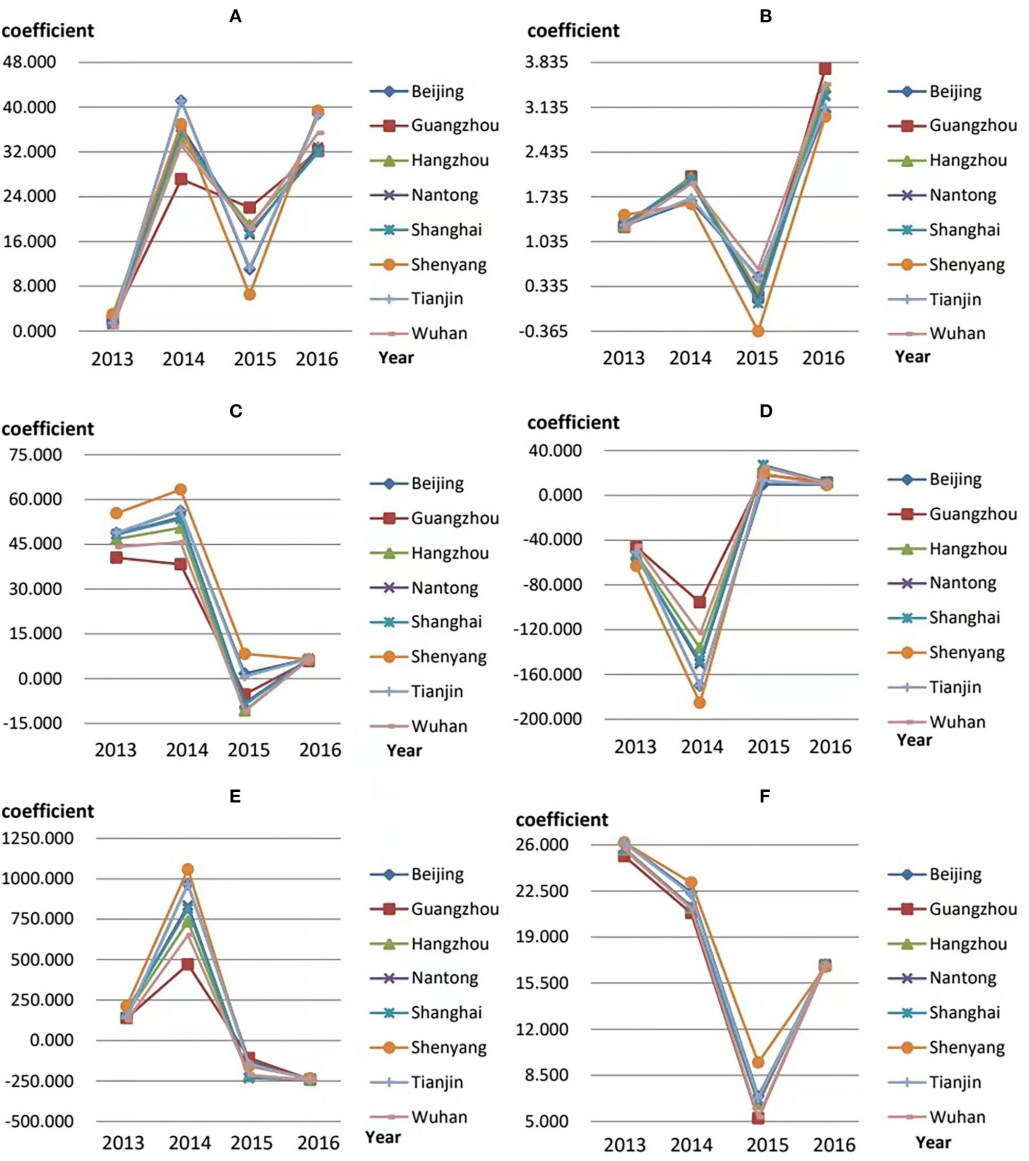
Temporal variation in the estimated coefficients. **(A–F)** represent the coefficient variations of LAN, PM2.5, per capita consumption expenditure, economic density, population density, and number of medical beds, respectively.

Figure 2 illustrates the variation in selected variable coefficients during 2013–2016 in Beijing, Guangzhou, Hangzhou, Nantong, Shanghai, Shenyang, Tianjin, and Wuhan (the scale of BC in the selected cities exceeds 1,000). In the Figures 2A–F denote LAN, PM2.5, per capita consumption expenditure, economic density, population density, and number of medical beds, respectively. In summary, the estimated coefficients of each variable in the selected cities have the same trends over time, which can be divided into two categories. One is the three-stage fluctuation mode, namely, the trend of coefficients in each city from 2014 to 2015 is opposite to that in the previous and subsequent periods, and (A), (B), (C) and (D) all show this characteristic. Taking LAN as an example, its fluctuation feature over 4 years is “rise-fall-rise,” and the positive impact fluctuates repeatedly. It is worth mentioning that the economic density has an opposite impact on the scale of BC. In 2013–2014 its increase effectively reduced the scale of BC, but in the next 2 years, it showed the promotion to the scale of BC. The other is the two-stage fluctuation mode. The change in the coefficient of population density and the number of medical beds over time is consistent with this model. The former showed a rise followed by a fall, while the latter showed the opposite. The trend of population density changes from a strengthening promoting effect to a strengthening inhibiting effect. In contrast, the impact of the number of medical beds is always positive and decreases year by year until 2015, only to pick up in 2016. In conclusion, the inclusion of time effects can reflect the influencing trend of various factors and be beneficial to clarify the direction and focus of BC prevention.

### Spatial variation of estimated coefficients

To show the effect of each factor on the spatial distribution of the BC scale more clearly, we will describe them through the spatial distribution characteristics map.

**Figure 3:**
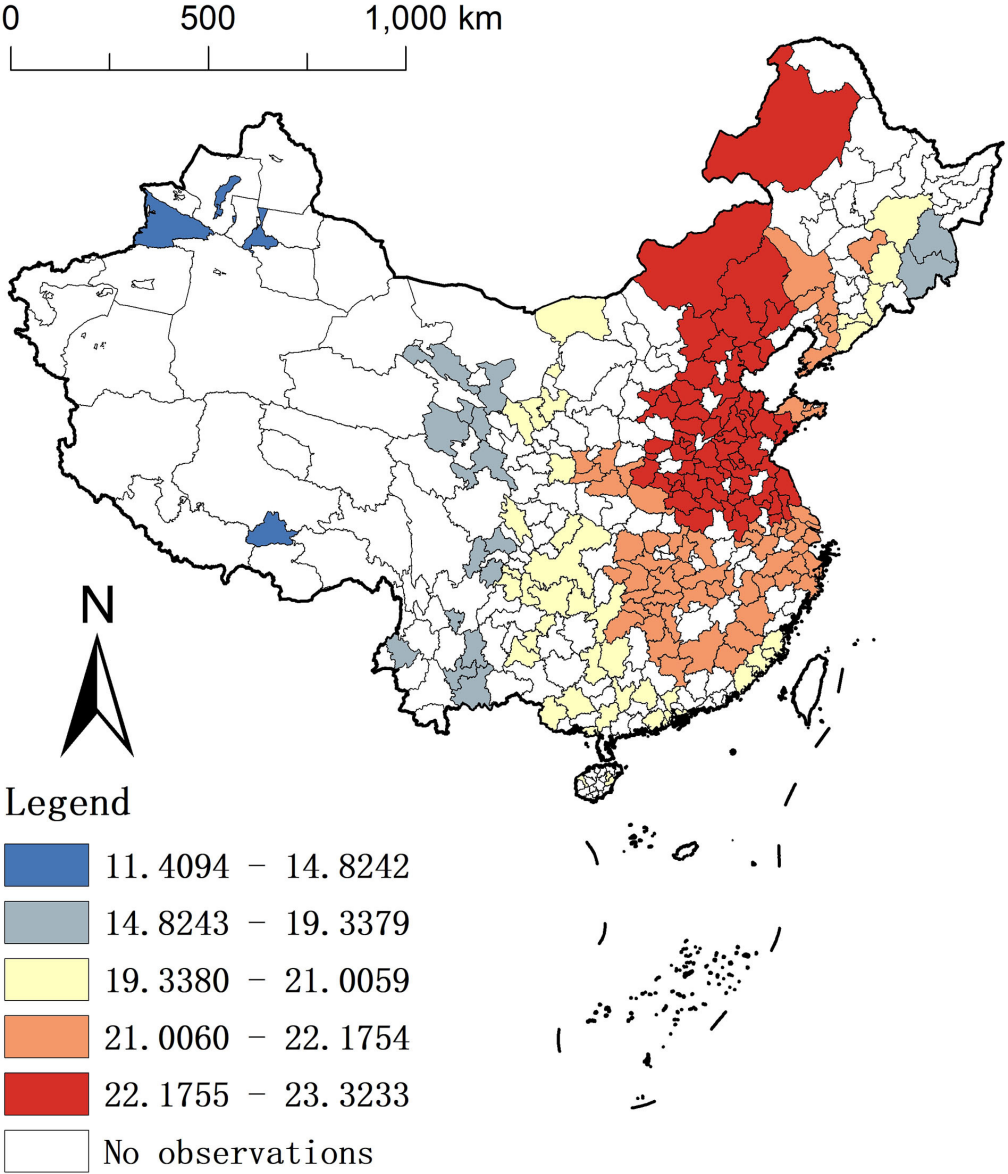
Spatial distribution of the average coefficients for LAN.

As shown in Figure 3, the average coefficient for LAN displays a pattern in which “the positive correlation increases from west to east.” In other words, the positive impact of LAN on the BC scale is strengthening in eastern China but weaker in western China, which shows consistency with our hypothesis.

**Figure 4:**
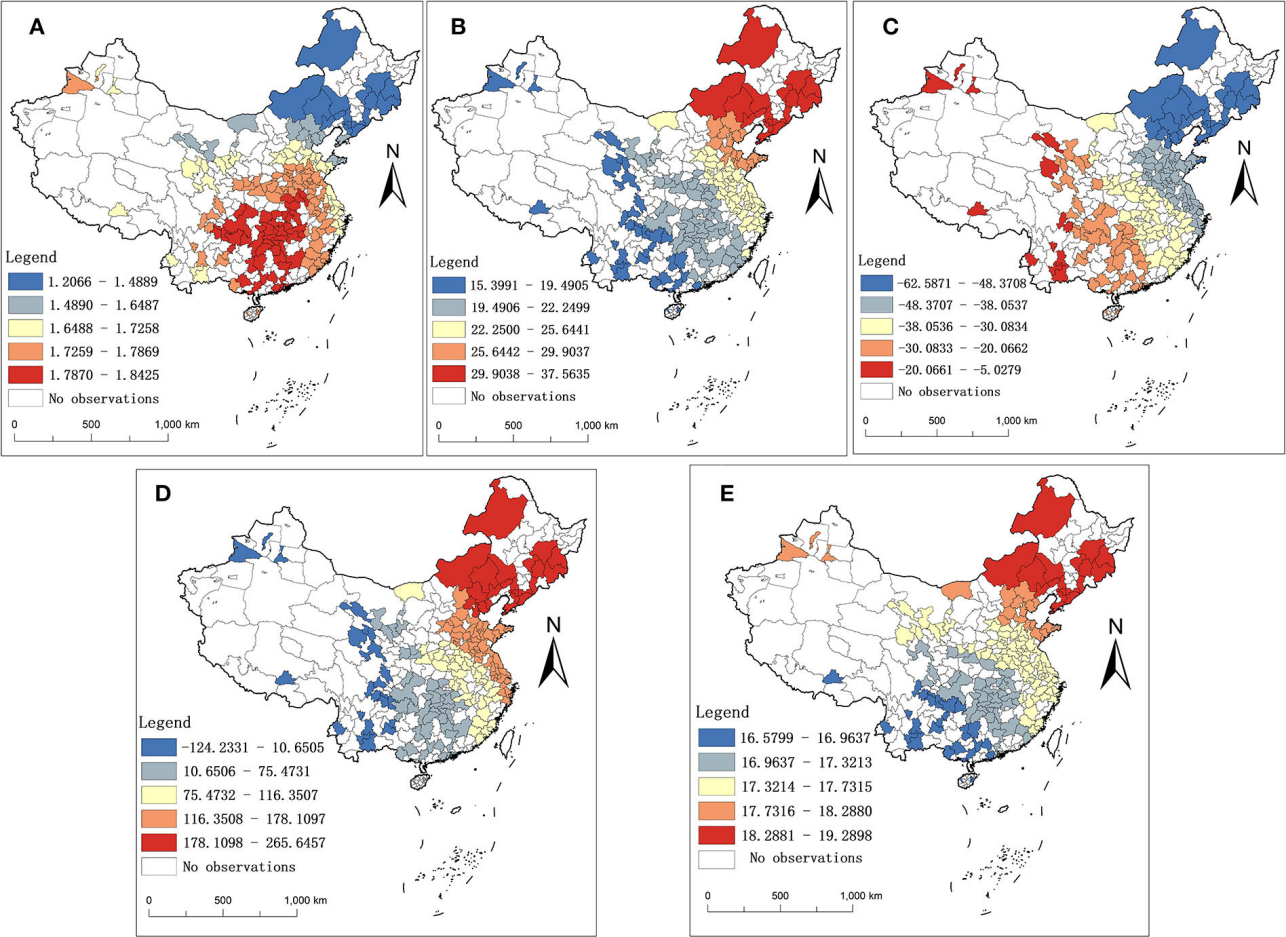
Spatial distribution of the average coefficients for other explanatory variables. **(A–E)** represent the spatial distribution of the average coefficients of PM2.5, per capita consumption expenditure, economic density, population density, and number of medical beds, respectively.

In Figures 4A–E represent the spatial distribution of the average coefficients of the GTWR model for the five explanatory variables, PM2.5, per capita consumption expenditure, economic density, population density, and the number of medical beds, respectively, during 2013–2016. According to the spatial patterns, we can summarize them into three forms: (1) Patterns in the north-south direction. The contribution of PM2.5 to the BC scale increases from north to south. In most southern cities, the BC scale was more positively influenced by PM2.5 concentrations, with regression coefficients ranging from 1.79 to 1.84 in the highest rank. (2) The degree of the positive impact shows an increasing pattern from west to east. The variables of per capita consumption expenditure, population density, and number of medical beds all fall into this category. In other words, the BC scale increases less with increasing per capita consumer spending, population density, and number of medical beds in western cities relative to those in the east. (3) The degree of the negative impact also shows an increasing pattern from west to east. The coefficient of economic density is consistent with this pattern. Concretely, a one-unit increase in economic density reduces the BC scale more in eastern cities than in western cities. In addition, the results of population density and economic density in the GTWR model are contrary to the previous OLS, which illustrates the necessity to consider the spatial perspective.

## Discussion

Our study has important implications. First, China is the largest developing country and is representative of the vast regional disparities in economic development and the clustering status of the BC scale. The corresponding policy recommendations can provide lessons for other regions with high BC pathogenesis. Second, compared with the classical regression models (e.g., OLS, GWR, and TWR), the GTWR model, shows the best performance in studying the problem of this study. This result proves the validity of the GTWR model in modeling the spatiotemporal heterogeneity at the BC scale. It also precisely portrays the spatiotemporal leap trajectory of the impact of each explanatory variable on the BC scale, mainly LAN (60). Third, this study focused on the macro factors of the BC scale. It is an innovative exploration compared with previous studies that investigated only from the biogenetic perspective. This study captured the characteristics of macro elements based on their influence on the BC scale. Additionally, it provides an evidence-based foundation for the differentiated implementation of regional health policies. At the same time, it strengthens the government's initiatives in the two dimensions of time and space.

### How can governments break the stalemate over the timing of the rise in the BC scale?

The gradual upward trend of the BC scale has intensified over time. From the perspective of governments, to curb or mitigate this trend, they should first focus on the macro factors that affect the BC scale. Governments should not only continue to promote the development of protective factors but also try to curb the deterioration and recurrence of risk factors over time.

First, LAN and PM2.5 always appear to be risk factors for BC. The coefficients for both show an overall increasing time trend (except in 2015), indicating that they have a growing degree of influence on the scale of BC. Therefore, the government needs to take initiatives to reduce overall LAN and PM2.5 in the city, minimize large-scale forms of nighttime operations and continue to impose strict regulations on factory exhaust and vehicle emissions. Second, per capita consumption expenditure and economic density have changed from risk factors to protective factors over time. Spending power and economic density increases indicate that the population is wealthier, and disease prevention is more effective among them (61), which can explain its suppressive impact on BC. Third, the positive impact of the number of medical beds and population density played a more prominent role in increasing the BC scale in 2015–2016. Specifically, the dangerous degree of the number of medical beds first decreased and then rebounded in 2016. Due to the progress of society, abundant medical resources have led to a significant increase in cancer detection rates. In addition, the improvement of medical testing and the enhancement of people's health awareness have played a vital role in slowing down the occurrence of diseases. However, the situation may be worse (62, 63) if we do not consistently reinforce the level of medical care and health awareness. Population density manifested as a transition from protective factors to risk factors. The increasing life expectancy of the elderly and the liberalization of the two-child policy in 2016 have brought a new round of increase in population density. The increase in the population base leads to an increase in population density, which in turn expands the BC scale. If the government can continue to optimize the “birth policy” and “pension policy,” the development of a healthy population structure in China is just around the corner, and the problem of population density will be solved.

### How can governments respond to the spatial heterogeneity of the BC scale?

In epidemiological studies, **in addition to** people and time, location is also a vital dimension (64). The reasons for the heterogeneity in the spatial distribution of the BC scale are diverse (34, 65). Therefore, the government also needs to change the spatial heterogeneity pattern of the BC scale based on multiple macro perspectives and different regions; otherwise, it will be challenging to achieve a breakthrough in “restraining the rapid growth of the BC scale” in the short term.

1. Considering that lights can directly reflect the local industrialization level, urbanization level, and population concentration (66), its distribution pattern is consistent with China's economic development gradient from west to east, which is relatively reasonable. There is no doubt that LAN extends our leisure, entertainment, office, and study time and makes great contributions to improving our quality of life. However, this is also a hazard. It limits the brightening effect of the stars at night. Additionally, it artificially increases energy consumption, breaks the balance of the natural environment, increases the BC scale, and even seriously damages human life and health. How can we, with government's help, both enjoy the fun of LAN and reduce its health hazards? The eastern region, where the coefficient of LAN is higher, should pay more attention to the management of LAN. The government should focus on setting the lighting source control and lighting limit technical requirements in some developed eastern cities. For example, mitigation can be achieved by investigating the actual needs to provide the minimum lighting level required for walking, driving, and by setting street lights to avoid targeting residential areas. In addition, local governments should implement zoning management for residential, commercial, traffic road, industrial, and landscape areas according to their development (67) and reduce lighting in public spaces to the lowest acceptable level. Furthermore, it is necessary to improve the transparency of information. In the current situation where people lack knowledge about the hazards of light pollution, the government should monitor health warnings and other content on the sales packaging of lighting objects to protect consumers' right to know. Finally, it is worth mentioning that although light pollution control methods emerge in an endless stream, the control process also requires the government to comprehensively consider the local population size, economic development, characteristics of human health development, and the carrying capacity of the medical and health system.
2. High regression coefficients of PM2.5 were largely concentrated in southern cities of China. Many developed enterprises will emit waste gas because of the pursuit of rapid development. In recent years, although China has strictly monitored the goals of “energy saving, emission reduction, and emission standards,” there are still “fish that slip through the net.” Moreover, low rainfall and wind speed in winter, exogenous imported pollution from northern cities, and high motor vehicle exhaust (68, 69) all contribute to the concentrations of PM2.5 and further lead to higher human breast density and consequently a larger BC scale. Therefore, the southern regions' governments should speed up air pollution management. In the face of air pollution caused by enterprises, it is necessary to carefully approve the site selection of the enterprise, especially the location upwind of the city, and to increase the cost of exhaust emissions through environmental protection tax, etc., and encourage enterprises to eliminate outdated processes and equipment and use more clean energy such as wind energy and solar energy. It is also crucial for the government to continuously strengthen green management in construction as well as vehicle transport management (70, 71). In addition, the government should call on people to prepare necessary protective equipment outdoors, such as masks, to reduce the harm of some inhalable particles.
3. The regression coefficients of per capita consumption expenditure, population density, number of medical beds, and economic density all have significant regularity in the east-west direction. The specific performance is that the promotion effect of the first three is gradually increasing from west to east, and the latter is the opposite. This reflects, for one thing, the drawbacks of excessive economic development in developed regions, such as people's anxiety due to more significant life stress (72), leading to a greater degree of influence of various risk factors. In addition, it reflects the imbalance of economic resources, population size, and medical resources between the eastern and western regions of China. Therefore, local governments cannot simply pursue economic benefits at the expense of regional population health. The development process of China's eastern and western regions is relatively complex, and the two are at different stages of economic development. The western region is limited by harsh climate and terrain conditions, and the development of various fields lags. However, the eastern region has entered a new stage of focusing on individual characteristics by mapping high technology in the medical field and gathering medical talent. Therefore, the developed eastern region should pay more attention to mental health, while the western region needs to invest in medical resources to alleviate the rapid increase in the BC scale. The government can make significant progress in BC prevention by eliminating the “one size fits all” policy approach across the country.

### Limitations

This study also had some limitations. The spatial units involved in this study include only 182 prefecture-level cities, which may lead to insufficient spatial non-stationarity. In larger spatial regions, spatial heterogeneity features are usually more significant. In addition, based on the availability of data, the BC scale was used as the dependent variable instead of using BC morbidity. Although we added population density as an explanatory variable to the model later, there are still limitations in describing the morbidity status of BC. Last, due to the constraints of data and spatial research methods, our explanatory variables do not include micro-level influencing factors such as lifestyle and genetic inheritance, nor do we have macro-level variables covering all fields to achieve a perfect fit with reality. In the future, we will still pay further attention to the shortcomings of these aspects, with the aim of providing a more detailed and realistic description of the effects, the scope of influence, and the degree of influence on BC.

## Conclusion

This study evaluated the spatial and temporal associations between the scale of BC and macroscopic factors in 182 prefectural Chinese cities by using the GTWR model. Regardless of the time dimension or the space dimension, the macro factors show obvious differences. If the government cannot take differentiated and targeted measures based on multiple perspectives and different regions, this will seriously restrict the integration of health into all policies. Additionally, it will also make people in different regions lack equity in BC prevention, further exacerbating the unequal development of the region.

We put forward some targeted policy recommendations. First, the control of LAN should focus on the developed cities in the east, especially to set the lighting source control and lighting limit technical requirements and to warn consumers of light hazards. Second, the control of environmental pollutants such as PM2.5 should be led by southern cities. Not only must strict requirements be placed on the source of pollutant emissions, such as restrictions on exhaust emissions from enterprises and automobiles but also the concentration of pollutants must be reduced by increasing the green area. The eastern region in the mature stage of economic development should focus on individual situations, such as immense psychological pressure. The western region, which is relatively lagging in economic development, should focus on economic development and be ready to undertake the transfer of developed medical technology from the eastern region. Last but not least, policies formulated by the government on strengthening economic development and consumption capacity or weakening LAN, PM2.5, etc., must ensure the continuity of time, and continue to progress in the process of “implementation-optimization.”

## Data availability statement

The original contributions presented in the study are included in the article/supplementary material, further inquiries can be directed to the corresponding authors.

## Author contributions

XB is responsible for writing original draft and revision. XZ is responsible for visualization. HS, YLi, and YC are responsible for writing review and editing. GG, BW, YLa, WX, and YW are responsible for data collection and literature retrieval. BS is responsible for framework design and supervision. YLi is responsible for supervision. All authors contributed to the article and approved the submitted version.

## Funding

This work was supported by the Humanities and Social Sciences Foundation of the Ministry of Education of China (Grant No. 19YJCGAT004), National Social Science Foundation of China (Grant No. 20BGJ026), the project “Culture, Port Culture and the Land Port Areas in Consolidating the Sense of Community for the Chinese Nation” by Minzu University of China (MUC) (Grant No. 2021MDZL15), National Ethnic Affairs Commission of the People's Republic of China 2022 Project - A study on the Differentiation of modernization policies in Ethnic Minority areas, and National Natural Science Foundation (72174047 and 71874045).

## Conflict of interest

The authors declare that the research was conducted in the absence of any commercial or financial relationships that could be construed as a potential conflict of interest.

## Publisher's note

All claims expressed in this article are solely those of the authors and do not necessarily represent those of their affiliated organizations, or those of the publisher, the editors and the reviewers. Any product that may be evaluated in this article, or claim that may be made by its manufacturer, is not guaranteed or endorsed by the publisher.

## Abbreviations

BC: Breast Cancer
LAN: Light at Night
GTWR: Geographic and Time Weighted Regression
IARC: International Agency for Cancer Research
VIIRS/DNB: Visible Infrared Imaging Radiometer Suite Day/Night Band
CNRDS: Chinese Research Data Services Platform
OLS: Ordinary Least Squares
GWR: Geographically Weighted Regression
TWR: Time Weighted Regression.
